# Metabolic syndrome amongst adults in Jordan: prevalence, trend, and its association with socio-demographic characteristics

**DOI:** 10.1186/s13098-020-00610-7

**Published:** 2020-11-18

**Authors:** Kamel Ajlouni, Yousef Khader, Mohamad Alyousfi, Mohannad Al Nsour, Anwar Batieha, Hashem Jaddou

**Affiliations:** 1 The National Center for Diabetes, Endocrinology and Genetics, The Jordan University, Amman, Jordan; 2 Department of Public Health, Jordan University of Science and Technology, P.O.Box 3030, Irbid, 22110 Jordan; 3 Department of Public Health, Faculty of Medicine, Jordan University of Science & Technology, Irbid, Jordan; 4 The Eastern Mediterranean Public Health Network (EMPHNET), Amman, Jordan; 5 Department of Public Health, Jordan University of Science and Technology Irbid, Irbid, Jordan

**Keywords:** Metabolic syndrome, Diabetes, Obesity, Hypertension

## Abstract

**Background:**

Multiple epidemiological studies were conducted amongst a variety of ethnic groups and showed discrepancies in the prevalence of metabolic syndrome (MeS) and its individual components. This study aimed to determine the prevalence of MeS in Jordan using both the Adult Treatment Panel Guidelines (ATP III) and the International Diabetes Federation (IDF) criteria. The study also aimed to assess the changes in the prevalence of MeS over time and determine its association with sociodemographic variables.

**Methods:**

Data from the 2017 Cardiovascular Disease Risk Factors Survey were used for this study. Socio-demographic and clinical data were collected using a structured questionnaire. Blood samples were taken for biochemical measurements. Furthermore, anthropometric characteristics were measured by the same team of trained field researchers. A sample of 4,056 individuals aged between 18 and 90 years was included in this study. The findings from the 2017 survey were compared with the findings of a 2009 survey that adopted the same methods and procedures.

**Results:**

According to the IDF criteria, the crude prevalence of Metabolic syndrome was 48.2% (52.9% among men and 46.2% among women; p < 0.001). Using the ATP III criteria, the prevalence was 44.1% (51.4% among men and 41% among women, p < 0.001). The age-standardized prevalence rate of metabolic syndrome was 44% (95% CI 42.7, 45.4) and 39.9% (95% CI 39.6, 41.2), according to both the IDF definition and ATP III criteria, respectively. The Kappa measure of agreement showed excellent agreement between the two definitions (k = 81.9%, p < 0.001). Of all participants, 41.7% met both the IDF and ATP III diagnostic criteria, 6.6% met the IDF criteria only, and 2.5% met the ATP III criteria only. The age-standardized prevalence rate of metabolic syndrome was significantly higher in 2017 (45.7% in men and 44.5% in women) than that in 2009 survey (34.6% in men and 39.8% in women). Gender, age, occupation, region, and marital status were significantly associated with metabolic syndrome.

**Conclusions:**

The prevalence of metabolic syndrome in Jordan is considerably high and it is increasing. Healthy lifestyle programs encouraging appropriate dietary habits and physical activity are strongly recommended in Jordan.

## Background

Non-communicable diseases (NCDs) are emerging as a global health concern with more remarkably increasing trends in low and middle-income countries [1]. The Global Burden of Diseases reports have shown that metabolic abnormalities are the most important determinants of NCDs [2]. MeS more strongly predicts cardiovascular diseases and increases the risk of developing diabetes mellitus and chronic kidney disease than its individual components do [3, 4]. There is an escalating concern regarding MeS in the past few years due to multiple factors such as epidemiologic transition, life-style changes, and the burden of nutrition disorders. However, confusion regarding MeS still exists due to the lack of a unified definition, the debates about its etiology and pathogenesis, and the lack of a consensus protocol for its treatment [2, 3, 5–7]. In 2006, the International Federation for Diabetes (IDF) demonstrated a standard definition for MeS as “a cluster of the most dangerous heart attack and cardiovascular diseases risk factors” [3]. This definition addresses both clinical and research needs, and it provides suitable and accessible tools for diagnosing MeS status.

The prevalence of MeS has been increasing worldwide for several decades especially in developing countries [8]. It is still hard to provide an accurate estimate of MeS prevalence due to the variety of definitions used. For instance, the prevalence ranges from 10–84% across different countries, ethnic groups, gender, and age groups. Multiple epidemiological studies were conducted among a variety of ethnic groups and showed discrepancies in the prevalence of MeS and its individual components [9, 10].

Jordan is a lower middle income country in the Eastern Mediterranean Region. According to the United Nations data, its population is estimated to be 10,203,134 as of mid-2020. In 2009, a survey was conducted in Jordan and showed that the age-standardized prevalence rate of MeS was 38.0% using the IDF criteria. The importance of studying the prevalence and trend of MeS stems from the fact that it could potentially be used as an adequate index for detecting people who are at a high risk of NCDs and other cardiovascular diseases [2]. Therefore, a similar survey was conducted within the same population in 2017. This study aimed to determine the prevalence of MeS in Jordan using the Adult Treatment Panel III (ATP III) and IDF criteria, to assess the changes of MeS prevalence over time, and to determine its association with sociodemographic variables.

## Methods

### Study design and sampling

A survey was conducted among Jordanian adults over a period of four months in the year 2017. The survey methods and procedures were described and detailed in other publications [11, 12] and they were similar to those that had been used in the previously conducted 2009 survey [13]. A multistage cluster sampling approach, adopting the probability proportional to size random selection method, was used to ensure the adequate coverage of the entire target population. A city/village was selected from each of the 12 governorates of Jordan. The sample of households was chosen in two stages. In the first stage, well-defined geopolitical areas (clusters) were selected from each city/village. At least one cluster was selected from each city/village randomly using computer-generated random numbers. The second stage of household selection involved choosing a random sample of households from a household list within a selected area. The households from each cluster were selected at random using a systematic sampling technique. A team of two persons (a female and a male) visited members of the selected households. After explaining the study to them, they were asked to visit the health center, while fasting on a given day. Subjects were also asked not to take their medications on the day of their visit and to bring them to the health center. Subjects aged ≥ 18 years were eligible for inclusion in the study. To encourage participation, the team worked on weekends and holidays and provided free transportation for those who asked for it.

The total sample size selected was 4056 participants with an overall response rate of 78.1%. Using this sample size and assuming that the prevalence of MeS is 50%, the power of the study to estimate the prevalence of MeS, within a margin of error of 5% at the alpha level of 0.05, exceeds 95%.

The study was approved by the Ethical Committee at the National Center for Diabetes, Endocrinology, and Genetics, Amman, Jordan (Ethical code: 1/2015). An informed consent was obtained from each participant. Data were treated with strict confidentiality and used only for scientific purposes.

### Data collection

Trained interviewers administered a comprehensive and structured questionnaire specifically prepared for the purpose of the study. The main data obtained included sociodemographic variables, diabetes, and other cardiovascular disease risk factors. Blood pressure was measured in a standardized way by trained researchers similar to what has been done in the 2009 survey [13]. Three blood samples were drawn from a cannula inserted into the antecubital vein and used for the different laboratory measurements. Tubes containing sodium fluoride potassium oxalate were used for glucose measurement. Samples were centrifuged within one hour at the survey site and then transferred by separate labeled tubes in iceboxes to the central laboratory of the National Center of Diabetes, Endocrinology, and Genetics in Amman, Jordan. All biochemical measurements were carried out by the same team of laboratory technicians and using the same method throughout the study period. Fasting plasma, glucose, and lipid measurements were performed according to the manufacturers’ instructions, using the COBAS autoanalyzer (Roche Diagnostics, Basel, Switzerland).

### Anthropometric measurements

Using digital scales (seca), the subjects’ weight was measured while they were minimally clothed and not wearing shoes. Their height was measured using a portable stadiometer (seca 214 portable stadiometer). Their Body Mass Index (BMI) was calculated as weight in kilograms divided by height in meters squared [14]. Their waist circumference (WC) was measured at the midway between the iliac crest and the lower rib margin, over light clothing, using un-stretchable tape (seca 203), and without any pressure to the body surface. Their Waist to Hip Ratio (WHR) was calculated as WC divided by hip circumference, and their waist to height (WHtR) was calculated as WC divided by height in centimeters. All measurements were taken by the same team of well-trained persons using the same tools.

### Definitions

Being overweight or obese was classified according to the definition of overweight (BMI of 25–29.9 kg/m^2^) and obesity (BMI of 30 kg/m^2^ or more) in adults [15]. MeS was defined according to IDF criteria and ATP III diagnostic criteria. Diabetes was defined according to IDF Diabetes Atlas 8th Edition Diagnostic Criteria [16].

### Statistical analysis

Data were entered and analyzed using the Statistical Package for Social Sciences Software “SPSS IBM version 24”. The raw data file for 2009 were re-analyzed using the same variable definitions to assess the time-trends in MeS prevalence. Proportions were used to estimate the prevalence of MeS. Overall and age-specific prevalence rates were obtained and reported separately for each gender. The age-standardized prevalence rates were derived in order to permit comparison between the different surveys and to allow comparison with studies in other countries, using the world population as a standard population. Chi-square was used to compare proportions. Multivariate binary logistic regression was conducted to determine factors associated with MeS. A p-value of less than 0.05 was considered statistically significant.

## Results

### Socio-demographic characteristics

This study included 4056 participants [1193 (29.4%) men and 2863 (70.6%) women] aged 18 years or more. About 26.5% of the sample were between 40 and 49 years old. Men were significantly older than women; the mean (SD) age was 47.5 (14.6) year for men and 42.2 (13.7) year for women (p < 0.001). The majority of participants (94%) were Jordanians while 6% were Syrians. Table 1 shows the socio-demographic characteristics of participants according to gender. The sample demographic breakdown (age, education, and nationality) is similar to national data.

**Table 1:** Socio-demographic, anthropometric and clinical characteristics of 4056 participants according gender, Jordan 2017

	Men (n= 1193) n (%)	Women (n= 2863) n (%)	Total (N=4056) n (%)	p –value*
Age (year)				< 0.001
18-29	155 (13)	582 (20.4)	737 (18.2)	
30-39	180 (15.1)	626 (21.9)	806 (19.9)	
40-49	313 (26.3)	758 (26.5)	1071 (26.5)	
50-59	294 (24.7)	557 (19.5)	851 (21)	
60-69	154 (12.9)	249 (8.7)	403 (10)	
≥ 70	95 (8)	84 (2.9)	179 (4.4)	
Marital status				< 0.001
Single	144 (12.1)	462 (16.1)	606 (14.9)	
Married	1049 (87.9)	2401 (83.9)	3450 (85.1)	
Nationality				0.942
Jordanian	1117 (94)	2669 (93.9)	3786 (93.9)	
Syrian	71 (6)	173 (6.1)	244 (6.1)	
Smoking status				< 0.001
Current	395 (33.1)	189 (6.6)	584 (14.4)	
Past	206 (17.3)	46 (1.6)	252 (6.2)	
Never	592 (49.6)	2628 (91.8)	3220 (79.4)	
	Mean (SD)	Mean (SD)	Mean (SD)	
Body mass index (kg/m^2^)	28.4 (4.8)	30 (6.4)	29.5 (6)	< 0.001
Waist circumference (cm)	98.9 (15.1)	92.7 (16.6)	94.6 (16.4)	< 0.001
Waist to hip ratio	0.94 (0.08)	0.85 (0.1)	0.88 (0.1)	< 0.001
Waist to height ratio	0.57 (0.08)	0.58 (0.1)	0.58 (0.1)	< 0.001
Systolic blood pressure (mmHg)	127 (19.6)	118.3 (19.7)	120.9 (20.1)	< 0.001
Diastolic blood pressure (mmHg)	79.5 (11.7)	75 (11.6)	76.3 (11.8)	< 0.001
Fasting blood glucose (mg/dL)	123.7 (58.6)	106.9 (41.6)	111.9 (47.9)	< 0.001
Total cholesterol (mg/dL)	193.3 (45.8)	197.5 (41.7)	196.2 (43)	< 0.05
HDL (mg/dL)	38.2 (9.5)	48.6 (12.3)	45.6 (12.5)	< 0.001
LDL (mg/dL)	125 (37.3)	126.9 (36.3)	126.3 (36.6)	0.135
Triglyceride (mg/dL)	203.8 (209.3)	147.3 (110.6)	164 (149)	< 0.001

* Chi-Square test is used to compare proportions between men and women and independent t test is used to compare means between men and women

### Anthropometric and clinical characteristics

The mean anthropometric and biochemical characteristics for Jordanians are shown in Table 1. There was a significant difference in the majority of studied parameters. The mean (SD) of BMI was 29.5 (6.0) kg/m², thus being significantly higher in women than in men (30 (6.4) kg/m² vs. 28.4 (4.8) kg/m²; *p* < 0.001). The mean systolic and diastolic blood pressure, mean fasting blood glucose, and mean triglyceride were significantly higher in men than in women. On the other hand, women showed significantly higher cholesterol, HDL, and LDL levels than men.

### The prevalence rate of MeS

The crude prevalence of MeS was 48.2% (52.9% among men and 46.2% among women; p < 0.001) according to the IDF criteria. According to the ATP III criteria, the prevalence was 44.1% (51.4% among men and 41% among women, p < 0.001). The age-standardized prevalence rate of MeS was 44% (95% CI 42.7%, 45.4%) and 39.9% (95% CI 39.6%, 41.2%) using the IDF definition and ATP III criteria, respectively. The kappa measure of agreement showed excellent agreement between the two definitions (k = 81.9%, p < 0.001). Of all participants, 41.7% met both the IDF and ATP III diagnostic criteria, 6.6% met the IDF criteria, and 2.5% met the ATP III criteria only. Tables 2 and 3 show the crude and age-standardized sex-specific prevalence rates of MeS and its individual components using the ATP III and IDF criteria.

**Table 2:** The sex-specific crude prevalence rates of Metabolic syndrome and its individual components in Jordan, using the ATP III and IDF definitions, Jordan 2017

	Men number (%)	Women number (%)	Total number (%)	P-value *
Metabolic syndrome				
IDF definition	625 (52.9)	1300 (46.2)	1925 (48.2)	< 0.001
ATP definition	608 (51.4)	1154 (41)	1762 (44.12)	< 0.001
Central obesity				
IDF definition	797 (67.3)	2193 (77.8)	2990 (74.6)	< 0.001
ATP definition	491 (41.5)	1772 (62.8)	2263 (56.5)	< 0.001
Body mass index				
Obesity	419 (36.1)	1355 (48.2)	1774 (44.7)	< 0.001
Overweight	478 (41.2)	819 (29.1)	1297 (32.7)	< 0.001
Elevated triglycerides	645 (54.5)	1031 (36.5)	1676 (41.8)	< 0.001
Low HDL	727 (61.5)	1644 (58.1)	2371 (59.1)	0.057
Elevated blood pressure	647 (54.5)	1077 (38.2)	1724 (42.9)	< 0.001
High fasting blood glucose				
IDF definition	560 (47.4)	938 (33.4)	1498 (37.5)	<0.001
ATP definition	476 (40)	692 (24.2)	1168 (28.8)	<0.001

* Chi-Square test is used to compare prevalence rates of Metabolic syndrome and its individual components between men and women

**Table 3:** Age standardized sex-specific prevalence rates of metabolic syndrome and its components in Jordan, using ATP III and IDF definitions, Jordan 2017

	Males	Females	Total
	Age-standardized rate (95% CI)	Age-standardized rate (95% CI)	Age-standardized rate (95% CI)
Metabolic syndrome			
IDF definition	45.7 (42.9, 48.5)	44.5 (43.0, 46.1)	44 (42.7, 45.4)
ATP III definition	43.9 (41.2, 46.5)	39.5 (37.9, 41.0)	39.9 (38.6, 41.2)
Central obesity			
IDF definition	60.4 (57.6, 63.2)	75.6 (74.2, 77.0)	70.9 (69.6, 72.3)
ATP III definition	36.5 (33.8, 39.3)	60.7 (59.1, 62.2)	52.7 (51.2, 54.1)
Body Mass Index			
Obesity	32.8 (30, 35.5)	46.2 (44.5, 47.9)	41.4 (39.9, 42.9)
Overweight	39.4 (36.3, 42.4)	28.8 (27.1, 30.5)	32.3 (30.8, 33.8)
Overweight and obesity	77.2 (69.4, 74.9)	74.5 (72.9, 76.0)	73.7 (72.4, 75.1)
Elevated triglycerides	50.1 (47.3, 53.1)	35.1 (33.4, 36.7)	38.5 (37.1, 39.9)
Low HDL	59.2 (56.2, 62.3)	57.4 (55.5, 59.2)	57.7 (56.1, 59.3)
Elevated blood pressure	46.7 (44.0, 49.5)	37.7 (36.2, 39.2)	39.8 (38.5, 41.1)
High fasting blood glucose			
IDF definition	39.2% (36.6, 41.8)	32.4% (30.9, 33.9)	34.2 (32.9, 35.5)
ATP III definition	32.1% (29.7, 34.5)	23.9% (22.5, 25.3)	26.1 (25, 27.3)

### Age-specific prevalence rate of MeS

**Fig. 1:**
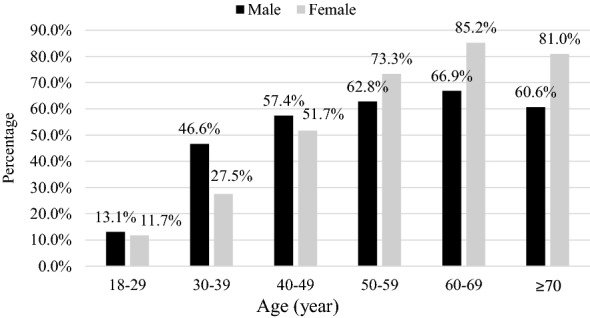
Age-specific prevalence of metabolic syndrome using the IDF definition in Jordan

**Fig. 2:**
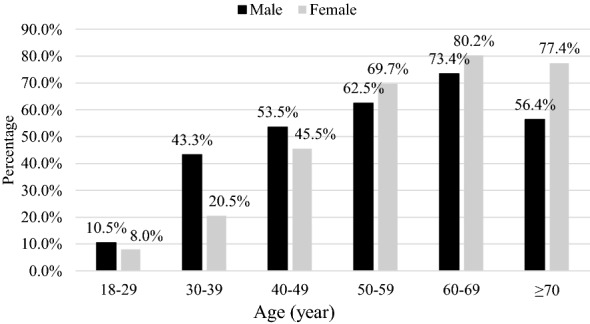
Age-specific prevalence of metabolic syndrome using the ATP III definition in Jordan

The age-specific prevalence of MeS for men and women is shown in Figs. 1 and 2 using the IDF definition and ATP III definition, respectively. According to both definitions, the prevalence of MeS increased significantly with age in both men and women (P < 0.001). With the IDF definition, the prevalence rose from 13.1% for men aged 18–29 years to 66.9% for those aged 60–69 years. Then, it declined to 60.6% in those aged 70 years and above. The prevalence rose from 11.7% in women aged 18–29 years to 85.2% in women aged 60–69 years. Then, it declined to 81.0% in those aged 70 years and above.

With the ATP III definition, the prevalence rose from 10.5% for men aged 18–29 years to 73.4% for those aged 60–69 years and then declined to 56.4% in those aged 70 years and above. While the prevalence rose from 8% in women aged 18–29 years to 80.2% in women aged 60–69 years, it declined to 77.4% in those aged 70 years and above.

### Prevalence of MeS syndrome according to socio-demographic characteristics

Tables 4 and 5 show the prevalence of MeS according to socio-demographic characteristics using the IDF and ATP III criteria, respectively.

**Table 4:** Prevalence of metabolic syndrome by age categories, smoking status, marital status, region and nationality among Jordanians using IDF definition, Jordan 2017

Variables	Men		*P* value*	Women		*P* value*
	No	Yes		No	Yes	
	Number (%)	Number (%)		Number (%)	Number (%)	
Age (years)			<0.001			<0.001
18–29	133 (86.9)	20 (13.1)		506 (88.3)	67 (11.7)	
30–39	95 (53.4)	83 (46.6)		445 (72.5)	169 (27.5)	
40–49	132 (42.6)	178 (57.4)		364 (48.3)	390 (51.7)	
50–59	109 (37.2)	184 (62.8)		145 (26.7)	399 (73.3)	
60–69	51 (33.1)	103 (66.9)		36 (14.8)	207 (85.2)	
≥ 70	37 (39.4)	57 (60.6)		16 (19)	68 (81)	
Smoking status			<0.001			0.05
No	280 (47.6)	308 (52.4)		1413 (54.6)	1174 (45.4)	
Past	68 (33.3)	136 (66.7)		20 (43.5)	26 (56.5)	
Current	211 (53.8)	181 (46.2)		81 (43.5)	105 (56.5)	
Marital status			<0.001			<0.001
Single	126 (89.4)	15 (10.6)		380 (83.3)	76 (16.7)	
Married	433 (41.5)	610 (58.5)		1134 (48)	1229 (52)	
Region			<0.001			<0.001
North	179 (46)	210 (54)		459 (50.4)	451 (49.6)	
Middle	261 (55.8)	207 (44.2)		753 (58.5)	534 (41.5)	
South	119 (36.4)	208 (63.6)		302 (48.6)	320 (51.4)	
Nationality			0.045			0.043
Jordanian	517 (46.7)	591 (53.3)		1422 (54.2)	1204 (45.8)	
Syrian	41 (57.7)	30 (42.3)		81 (47.1)	91 (52.9)	

* Chi-Square test is used to compare prevalence rates of Metabolic syndrome according to studied variables for men and women

**Table 5:** Prevalence of metabolic syndrome by age categories, smoking status, marital status, region and nationality among Jordanians using ATP III definition, Jordan 2017

Variables	Men		*P* value*	Women		*P* value*
	No	Yes		No	Yes	
	Number (%)	Number (%)		Number (%)	Number (%)	
Age (years)			<0.001			< 0.001
18-29	137 (89.5)	16 (10.5)		527 (92)	46 (8)	
30-39	101 (56.7)	77 (43.3)		488 (79.5)	126 (20.5)	
40-49	144 (46.5)	166 (53.5)		411 (54.5)	343 (45.5)	
50-59	110 (37.5)	183 (62.5)		165 (30.3)	379 (69.7)	
60-69	41 (26.6)	113 (73.4)		48 (19.8)	195 (80.2)	
≥70	41 (43.6)	53 (56.4)		19 (22.6)	65 (77.4)	
Smoking status			<0.001			< 0.05
Never	295 (50.2)	293 (49.8)		1541 (59.6)	1046 (40.4)	
Past	76 (37.3)	128 (62.7)		24 (52.2)	22 (47.8)	
Current	205 (52.3)	187 (47.7)		95 (51.1)	91 (48.9)	
Marital status			<0.001			< 0.001
Single	126 (89.4)	15 (10.6)		398 (87.3)	58 (12.7)	
Married	450 (43.1)	593 (56.9)		1262 (53.4)	1101 (46.6)	
Region			<0.001			< 0.001
North	169 (43.4)	220 (56.6)		517 (56.8)	393 (43.2)	
Middle	267 (57.1)	201 (42.9)		821 (63.8)	466 (36.2)	
South	140 (42.8)	187 (57.2)		322 (51.8)	300 (48.2)	
Nationality			<0.001			0.081
Jordanian	527 (47.6)	581 (52.4)		1555 (59.2)	1071 (40.8)	
Syrian	48 (67.6)	23 (32.4)		92 (53.5)	80 (46.5)	

* Chi-Square test is used to compare prevalence rates of Metabolic syndrome according to studied variables for men and women

### Prevalence rate of individual components of MeS

**Fig. 3:**
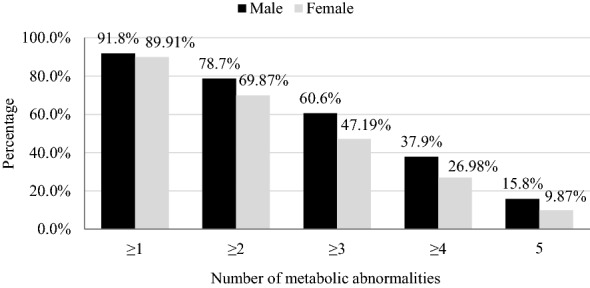
The relative frequencies of the number of metabolic abnormalities according to IDF definition

**Fig. 4:**
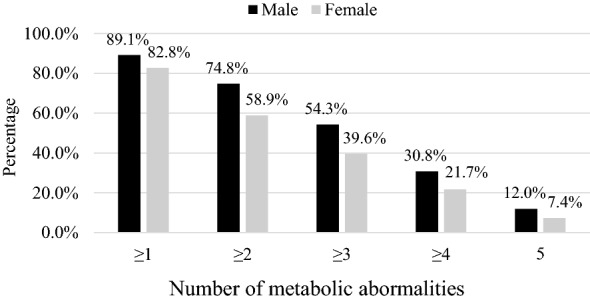
The relative frequencies of the number of metabolic abnormalities according to ATP III definition

The prevalence of obesity in Jordan was 44.7% and it was significantly higher in women (48.2%) compared to men (36.1%) (p < 0.001). The age-standardized prevalence of obesity was 41.4% (95% CI 39.9%, 42.9%). Among components of MeS, abdominal obesity was the most prevalent metabolic abnormality. According to the IDF definition, women had a higher prevalence of abdominal obesity (77.8%) compared to men (67.3%) (p < 0.001). Low HDL cholesterol was the second most common metabolic abnormality in both men and women. Elevated Triglycerides and low HDL level prevalence rates were 41.8% and 59.1%, respectively. The difference between men and women was statistically significant in elevated triglycerides level (p < 0.001) but not in low HDL level (p = 0.057). High fasting blood sugar prevalence rates were 37.5% and 28.8% according to both the IDF and the ATP III definitions. Men recorded a significantly higher prevalence of high fasting blood sugar using both the IDF and ATP III definitions (47.4% and 40% respectively) compared to women (33.4% and 24.2% respectively). The relative frequencies of the number of metabolic abnormalities according to the IDF and the ATP III measurements are shown in Figs. 3 and 4.

### Changes in the MeS Rate between 2009 and 2017

The 2017 sample had a higher mean age compared to the 2009 sample (43.8 (14.2) year and 41.8 (13.3) years, respectively). A comparison in the sex-specific age-standardized prevalence rates of MeS between 2009 and 2017 surveys is shown in Table 6. The age-standardized prevalence rate of MeS was significantly higher in 2017 (45.7% in men and 44.5% in women) than in 2009 survey (34.6% in men and 39.8% in women). Among men and women, the age-standardized rates of abdominal obesity and high fasting blood glucose were higher in 2017 than those in 2009. The 2009 survey showed higher prevalence rates of elevated blood pressure, elevated triglycerides, and low HDL levels.

**Table 6:** The sex-specific age-standardized prevalence rates of metabolic syndrome according to IDF definition and its individual components in two different periods (2009 and 2017), Jordan

	2017	2009
	Age-standardized prevalence rate (95% CI)	Age-standardized prevalence rate (95% CI)
Metabolic syndrome		
Male	45.7 (42.9, 48.5)	34.6 (31.4, 37.8)
Female	44.5 (43.0, 46.1)	39.8 (38.1, 41.5)
Total	44 (42.7, 45.4)	38.0 (36.5, 39.5)
Central obesity		
Male	60.4 (57.6, 63.2)	41.5 (38.0, 45.1)
Female	75.6 (74.2, 77.0)	60.1 (58.2, 61.9)
Total	70.9 (69.6, 72.3)	55.2 (53.5, 56.9)
High fasting blood glucose		
Male	39.2 (36.6, 41.8)	28.7 (25.8, 31.6)
Female	32.4 (30.9, 33.9)	23.9 (22.3, 25.5)
Total	34.2 (32.9, 35.5)	25.1 (23.7, 26.4)
Elevated blood pressure		
Male	46.7 (44.0, 49.5)	47.2 (43.5, 50.8)
Female	37.7 (36.2, 39.2)	38.6 (37.0, 40.3)
Total	39.8 (38.5, 41.1)	40.2 (38.7, 41.6)
Elevated triglycerides		
Male	50.1 (47.3, 53.1)	54.4 (50.4, 58.3)
Female	35.1 (33.4, 36.7)	40.0 (38.1, 42.0)
Total	38.5 (37.1, 39.9)	42.7 (41.0, 44.4)
Low HDL		
Male	59.2 (56.2, 62.3)	70.0 (66.1, 73.8)
Female	57.4 (55.5, 59.2)	64.7 (62.7, 66.7)
Total	57.7 (56.1, 59.3)	65.7 (64.0, 67.5)
HDL high-density lipoprotein		

### Factors associated with MeS

A multivariate logistic regression analysis was conducted to determine factors associated with MeS (Tables 7 and 8). Defined by the IDF diagnostic criteria, many factors were associated with MeS including gender, age, occupation, region, and marital status.

**Table 7:** Multivariate logistic regression model of the factors associated with metabolic syndrome, using the IDF criteria, Jordan 2017

	OR (95% CI)	P-value*
Gender		
Men	1 (Ref)	
Women	1.42 (1.10, 1.83)	< 0.05
Age (years)		
18–29	1 (Ref)	< 0.001
30–39	2.53 (1.79, 3.57)	< 0.001
40–49	5.78 (4.16, 8.03)	< 0.001
50–59	12.44 (8.73, 17.73)	< 0.001
60–69	14.54 (9.42, 22.42)	< 0.001
≥70	10.45 (6.26, 17.44)	< 0.001
Occupation		
Field Work	1 (Ref)	< 0.05
Unemployed	1.38 (1.02, 1.86)	< 0.05
Retired	0.95 (0.67, 1.35)	0.758
Office Work	1.01 (0.76, 1.36)	0.925
Region		
North	1 (Ref)	< 0.001
Middle	0.66 (0.54, 0.80)	< 0.001
South	1.14 (0.90, 1.44)	0.282
Marital status		
Single	1 (Ref)	
Married	2.26 (1.61, 3.18)	< 0.001

* p-values for adjusted odds ratios from multivariate binary logistic regression

**Table 8:** Multivariate logistic regression model of the factors associated with metabolic syndrome, using the ATP III criteria, Jordan 2017

	OR (95% CI)	P value*
Age (years)		
18–29	1 (Ref)	< 0.001
30–39	2.65 (1.80, 3.90)	< 0.001
40–49	7.00 (4.85, 10.11)	< 0.001
50–59	17.11 (11.60, 25.22)	< 0.001
60–69	20.62 (13.00, 32.72)	< 0.001
≥70	12.20 (7.18, 20.71)	< 0.001
Occupation		
Field Work	1 (Ref)	< 0.001
Unemployed	1.76 (1.32, 2.35)	< 0.001
Retired	1.17 (0.81, 1.68)	0.400
Office Work	1.14 (0.84, 1.54)	0.400
Region		
North	1 (Ref)	< 0.001
Middle	0.63 (0.52, 0.76)	< 0.001
South	1.10 (0.86, 1.40)	0.457
Marital status		
Single	1 (Ref)	
Married	2.00 (1.39, 2.89)	< 0.001

* p-values for adjusted odds ratios from multivariate binary logistic regression

Women had increased odds of MeS compared to men (OR = 1.42; 95% CI 1.1, 1.83,p < 0.05). Increased age was significantly associated with increased odds of MeS. Unemployed persons were more likely to have MeS (OR = 1.38; 95% CI 1.02, 1.86,p < 0.05) compared to those who had fieldwork. Compared to those who were living in the north of Jordan, those who lived in the mid-region of Jordan were less likely to have metabolic syndrome (OR = 0.66: 95% CI 0.54, 0.80, p < 0.001). Married people were more than twice (OR = 2.26) as likely to have Metabolic syndrome compared to singles.

## Discussion

This study reports the prevalence of MeS and its individual components among adults in Jordan using the IDF and the ATP III definitions and it compares the findings with the findings of a previous study conducted in 2009. Two diagnostic criteria were used to define MeS to facilitate the comparisons between the study findings and the findings of other studies that reported prevalence estimates using different definitions. The sample of Jordanians who participated in the study was representative of the general population of Jordan as the distribution of their relevant demographic factors was similar to that of the general population.

When the IDF diagnostic criteria was used, the age-standardized prevalence rates of MeS in Jordan was 44% (45.7% in men and 44.5% in women). This rate is slightly higher than what had been reported in the US population (40%) [17] and much higher than that in the Australian population (29.1%) [10]. The European population recorded rates from 10 to 30% [10], and the Iranian people recorded a rate of 37.4% [18]. Compared to Arab countries, the age-standardized prevalence of MeS was higher than the prevalence in Saudi Arabia in 2009 and 2014 (31.6% and 28.3%, respectively) [19, 20]. However, Aljabri et al. in 2018 reported a high prevalence rate (64.4%) among Saudi population [21]. The prevalence rates among the populations of Qatar and Kuwait were 37% and 36.2%, respectively [22, 23], while the MeS prevalence in the United Arab Emirates using the IDF criteria was reported in two studies conducted in 2008 and 2012 as 40.5% and 48.7%, respectively [24, 25]. The Lebanese population had a lower prevalence (31.2%) than that found in Jordan [26]. The prevalence in Jordan is very close to the prevalence reported among Turkish adults (44%) [18].

According to the ATP III criteria, the age-standardized prevalence rate of 39.9% (95% CI 38.6, 41.2) was lower than the prevalence defined by the IDF criteria. The two definitions almost have the same components, but the difference in prevalence was mainly linked to the difference in waist circumference. Abdominal obesity is considered an obligatory component for the IDF definition in contrast to being one of five components for the ATP III definition.

Compared to the US population, Jordan had a higher MeS prevalence than what had been reported in the US in 2014 (33.8%) using the ATP III criteria [27]. Also, it was higher than that in the Australian population (19.3%) [10], the Turkish population (36.6%) [18], and the European population (ranges from 10–30%) [10]. Compared to Arab countries, Jordan had a higher prevalence of MeS defined by the ATP III criteria than most Arab countries including Lebanon (26.4%) [28], Oman (23.6%) [29], Tunisia (31.2%) [30], the United Arab Emirates (22%) [31], Qatar (26.5%) [32], Yemen (23.8%) [33], and Kuwait (18.3%) [34].

On the other hand, two studies conducted in Saudi Arabia utilizing ATP III definition reported prevalence rates similar to that of Jordan. The first study in 2009 revealed a prevalence of 39.9% and an earlier study conducted in 2005 reported a prevalence of 39.3% [19, 35]. A study in the United Arab Emirates reported a prevalence of 50.3% in 2012 which is higher than Jordan’s prevalence rate [24]. Another study conducted in 2008 revealed a prevalence of 39.6% which is approximately similar to the rate found in this study [25]. The considerable discrepancy in the prevalence of MeS among and across different nations and populations could be a result of the integration of genetics, environmental aspects and factors, epidemiological transition, and differences in lifestyle. Differences in the definition used and differences in the sampling approaches and procedures might also explain some of the variations in the prevalence rates [23–25].

Obesity and central obesity may have an effect on this variation across different nations and populations, as central obesity is the most observed component among those diagnosed with MeS. Obesity increases the risk of developing multiple metabolic abnormalities including hypertension and insulin resistance which logically lead to developing MeS [36]. Consequently, the variation in the MeS prevalence between Jordan and other countries could be explained by the variation in obesity prevalence. In Jordan, the prevalence of obesity was 41.4% which is higher than what had been reported in Egypt (30.1%) [37], Lebanon (28.2%) [38], Syria (38.2%) [39], Saudi Arabia (33%) [40], United Arab Emirates (32.3%) [41], Qatar (35.4%) [42], Yemen (8.8%) [43], and Tunisia (25.4%) [44]. On the other hand, multiple studies in Saudi Arabia, Kuwait, and Libya revealed either higher or similar obesity prevalence rates compared to Jordan. Nadira Al-Baghli reported an obesity prevalence of 43.8% in Saudi Arabia [45]. The prevalence amongst Kuwaitis was estimated as 47.5% [23], while the prevalence was 42.4% in Libya [46].

The prevalence of MeS increased with age in both men and women, using the IDF and ATP III diagnostic criteria. The sharp increase happened during their second decade of life, especially for men. This could be explained by age-related changes in the body, insulin sensitivity, and fat distribution, all of which have been mentioned previously to contribute to the increased prevalence of MeS with age [47]. Women were observed to have higher MeS prevalence than men after their fourth decade of life. This continuously increasing prevalence in women could be a result of menopause. Menopause was reported to have an association with an increased risk of MeS and it is affecting all of its components [48]. On the contrary, the decrease in the prevalence among men and women after the sixth decade of life could be due to survival bias, where people affected by MeS die at a comparatively younger age, which leads to a depletion in the older age categories of affected individuals.

In our study, men had significantly higher MeS prevalence than women using both the IDF and ATP III diagnostic criteria. A study in Saudi Arabia supported our findings [19], while other studies did not [13, 20, 47]. The significant difference between men and women might be explained by age as men had a significantly higher mean age than women in this study. Age is strongly associated with an increased prevalence of MeS [10, 13, 27, 47]. Subsequently, after adjusting for age, occupation, location, and marital status, women had significantly increased odds of MeS compared to men only using the IDF. The reason might be that women have a significantly higher prevalence of abdominal obesity compared to men. On the other hand, data reported from the National Health and Nutrition Examination Survey (NHANES) among the US population from 2007 to 2014 did not show any significant gender differences [27].

For both men and women, abdominal obesity was the most prevalent component of MeS using the IDF and ATP III definitions. The prevalence rates of hyperglycemia, hypertriglyceridemia, and hypertension, despite being less common than abdominal obesity, are still high in this population. Women had a significantly higher crude and age-standardized prevalence rate of obesity using both the IDF and ATP III definitions. The explanation of the large waist circumferences and body mass index in women could be due to the fact that women in Jordan are less likely to participate in physical activity due to cultural and social limitations [47].

The age-standardized prevalence of MeS in this current study was markedly higher compared to a that in 2009 Jordan study. Also, the age-adjusted prevalence rates of abdominal obesity and hyperglycemia in this present population were higher than those in the 2009 population. On the other hand, lower age-standardized prevalence for low HDL levels was seen in a 2017 survey compared to the 2009 study. These variations in the prevalence could be explained by shifting from traditional dietary habits (a diet rich in fibers, vegetables, fruits, and cereals) to consuming more animal products and junk food, with high amounts of carbohydrates and saturated fats [13].

One of the main study limitations is the lack of available data on important predictors of MeS such as physical activity and dietary habits. Such variables are essential to be studied and to be included in the logistic regression model. Future research should consider such variables to determine the best predictors of MeS.

## Conclusions

The prevalence of MeS in Jordan is considerably high, and it is increasing. MeS was associated with gender, age, occupation, region, and marital status. This escalation in MeS prevalence is assumed to be a result of lifestyle changes and epidemiological transition, unhealthy dietary habits, and lack of exercise. Therefore, healthy lifestyle programs encouraging appropriate dietary habits and physical activity are strongly recommended in Jordan. Once the diagnosis is made, the potential treatment should be proactive and persistent in its goal of reducing the risk of CVD and type 2 diabetes.

## Data Availability

The data used to support the findings of this study are available from the corresponding author upon request.

## Abbreviations

NCDs: Non-communicable diseases
IDF: International Federation for Diabetes
ATP III: Adult Treatment Panel III
BMI: Body mass index
WC: Waist circumference
WHR: Waist to hip ratio
WHtR: waist to height ratio
HDL: High-Density Lipoprotein
LDL: Low-Density Lipoprotein
NHANES: National Health and Nutrition Examination Survey
